# Effect of Sulfated Polysaccharides and Laponite in Composite Porous Scaffolds on Osteogenesis

**DOI:** 10.3390/biom16010080

**Published:** 2026-01-03

**Authors:** Angelina Karamesouti, Maria Chatzinikolaidou

**Affiliations:** 1 School of Medicine, University of Crete, 70013 Heraklion, Greece; med12p1170051@med.uoc.gr; 2 Department of Materials Science and Engineering, University of Crete, 70013 Heraklion, Greece; 3 Institute of Electronic Structure and Laser, Foundation for Research and Technology-Hellas, 70013 Heraklion, Greece

**Keywords:** kappa-carrageenan, iota-carrageenan, pre-osteoblasts, bone tissue engineering, osteogenic differentiation

## Abstract

The design of biomaterial scaffolds for bone tissue engineering requires a balance between bioactivity, porosity, mechanical stability, and osteoinductivity. Kappa- (KC) and iota-carrageenan (IC) have been explored for scaffold fabrication due to their biocompatibility and structural similarity to glycosaminoglycans. However, there are limited reports on how their distinct sulfation degree affects the osteogenic differentiation of cells cultured on them. While laponite has been reported as an osteoinductive nanoclay, its combined effect with different carrageenan types and its concentration-dependent effect on scaffold functionality remain unexplored. Therefore, we developed composite scaffolds comprising poly(vinyl alcohol) (PVA) and gelatin (GEL), reinforced with kappa- or iota-carrageenan (KC, IC) and functionalized with two different concentrations of laponite (LAP), 0.5 and 1% *w*/*v*, to monitor composition-structure-function relationships. The scaffolds were fabricated via lyophilization and dual crosslinking, and characterized for their physicochemical, structural, mechanical, and biological properties. The incorporation of both carrageenans into scaffolds, maintained high swelling ratios of 600% after 24 h, and increased porosity without altering their apparent density (0.09–0.11 g/cm^3^), whereas LAP preserved interconnectivity, densified pore walls, raised their compressive modulus at >220 kPa, and improved stability (>60% mass retained after 40 days). In vitro validation using MC3T3-E1 pre-osteoblastic cells demonstrated robust cytocompatibility, with the LAP-containing scaffolds significantly promoting cell adhesion, proliferation, and osteogenic differentiation, evidenced by elevated alkaline phosphatase activity, calcium production and collagen secretion. Direct comparison between KC and IC scaffolds confirmed that differences in sulfate substitution modulated scaffold stiffness, swelling, and degradation, while variation in LAP concentration affected the biological response, with the 0.5 wt% concentration favoring early cell proliferation, whereas the 1 wt% significantly promoted the osteogenic differentiation. This compositional strategy demonstrates how tuning the interplay between carrageenan and laponite can balance scaffold hydration, mechanical and biological properties, thereby guiding the design of scaffolds for bone repair.

## 1. Introduction

For decades, one of the major challenges in medicine has been the development of substitute biological tissue materials capable of replacing, or regenerating, diseased, or damaged, tissues in patients [1]. Bone tissue engineering has emerged as a promising alternative to conventional bone grafts, employing functional biomaterials as scaffolds that mimic the extracellular matrix (ECM), providing a three-dimensional (3D) environment that supports cell adhesion, proliferation, and differentiation [2]. To this end, the properties of scaffolds are of utmost importance. Their chemical composition, biocompatibility, and osteoconductivity are essential to ensuring effective cancellous bone regeneration. Another crucial property is their porosity, as it facilitates cell penetration, vascularization and nutrient supply. Moreover, the degradation rate of the scaffolds should ideally match the rate of new tissue formation, enabling a gradual replacement by native bone. At the same time, mechanical stability is essential, as the scaffolds must withstand physiological stresses, comparable to natural bone [3,4].

In scaffold design for bone tissue engineering, biomaterials are broadly categorized into natural and synthetic ones. Natural biomaterials, such as proteins and polysaccharides, resemble the ECM, promoting cell adhesion and are biocompatible due to their low toxicity. Among them, gelatin (GEL), a denatured form of collagen, is widely used as it preserves bioactive sequences that support cell attachment and proliferation [5,6]. Additionally, sulfated polysaccharides, such as carrageenan, derived from red seaweed, have gained increasing interest in biomedical applications because of their gelling capacity, hydrophilicity, biocompatibility, and biodegradability [7,8]. These polysaccharides are naturally sulfated, and their biological functionality arises from intrinsic sulfate groups rather than from post-synthetic sulfonation processes, which have been extensively reviewed elsewhere [9]. Kappa-carrageenan (KC) and iota-carrageenan (IC) in particular are notable for their structural similarity to glycosaminoglycans (GAGs), enabling favorable biological interactions that support cell adhesion, proliferation, and differentiation [10,11,12]. Kappa-carrageenan contains one sulfate group per disaccharide unit, while iota-carrageenan has two sulfate groups, and these negatively charged groups promote electrostatic interactions with biomolecules, enhancing their biological functionality [8,13].

Nevertheless, natural polymers often lack adequate mechanical strength and long-term stability, making crosslinking or blending with other materials a necessity to improve scaffold robustness [14]. To address these limitations, synthetic polymers are often incorporated to enhance durability, processability, and enable controlled degradation, despite the fact that they may lack the inherent bioactivity of natural counterparts. Poly(vinyl alcohol) (PVA) is a linear, water-soluble, mechanically strong polymer, with high flexibility and elasticity [15]. Due to its abundance of hydroxyl groups, PVA exhibits a high swelling capacity in aqueous solutions, enabling it to absorb and retain biological fluids, which enhances cytocompatibility and supports scaffold integration with surrounding tissues [16].

Beyond polymeric systems, the incorporation of inorganic nanomaterials has attracted significant attention in tissue engineering, owing to their close resemblance to the mineral composition of bone tissue [3,16]. Materials such as hydroxyapatite [17], bioactive glasses [3], and calcium phosphates [18] have been widely explored as a means of reinforcing scaffold architecture and providing osteoinductive properties. Alongside them, laponite (LAP, Na^+0.7^[(Si^8^Mg^5.5^Li^0.3^)O^20^(OH)^4^]^−0.7^) [19], a synthetic layered silicate nanoclay, has recently emerged as a highly promising additive for bone tissue engineering [20]. It consists of disk-shaped nanoplatelets of a thickness between 1 and 2 nm and a diameter between 20 and 50 nm that feature a dual charge distribution, with negative surfaces and positively charged edges [21]. From a biological perspective, laponite displays remarkable bioactivity, largely due to the release of its constituent ions, which are magnesium (Mg^2+^), lithium (Li^+^), sodium (Na^+^), and silicate (Si(OH)_4_), and have been strongly associated with osteogenic differentiation, chondrogenesis, and extracellular matrix mineralization [22,23]. Several studies have demonstrated that laponite alone can stimulate osteogenesis of mesenchymal stem cells, even in the absence of additional osteoinductive factors [21,22]. These combined physicochemical and biological properties highlight laponite’s multifunctionality as a nanomaterial that reinforces scaffold architecture, while fostering cell-material interactions to support tissue repair.

The development of composite scaffolds that combine biomimetic biopolymers and bioactive nanomaterials has great potential for reproducing the hierarchical structure and functionality of bone. Kappa- and iota-carrageenans have been used individually [10,24] or combined within the same scaffold formulation [25], while comparative investigations report on how their different sulfation degree affects their physicochemical properties, gelation mechanism and network structure [26,27]. Moreover, the physicochemical properties of laponite and clay-containing composites have been previously reported [28,29]. Recent work on methacrylated gelatin, kappa-carrageenan bioinks incorporating laponite, reported on enhanced mechanical integrity and promoted osteogenic matrix deposition, supporting the rationale for hybrid carrageenan-nanoclay constructs [30].

Although carrageenans’ properties and applications have been explored, the effect of KC vs. IC on the scaffold architecture and their osteogenic differentiation capacity have not yet been validated. In addition, the effect of laponite combined with different carrageenan types has not been explored in the context of osteogenic scaffolds for bone tissue engineering. Hence, the hypothesis of this study is how carrageenan sulfation of KC and IC, and reinforcement with laponite affect the mechanical performance and osteogenic capacity of composite scaffolds. To address this hypothesis, scaffolds were reinforced with either KC or IC and functionalized with two laponite concentrations, 0.5 and 1% *w*/*v*. By comparing KC and IC, the study aimed to elucidate the effect of the sulfate substitution of the two carrageenans on the scaffold’s hydration, stability, and biological performance. In addition, the incorporation of two laponite concentrations aimed to assess its reinforcing role and capacity to provide osteoinductive cues through ion release. To this end, porous composite scaffolds were fabricated, and their physicochemical and mechanical properties were investigated. Structural characteristics, including apparent density, porosity, and compressive modulus, were quantified, alongside swelling and degradation, which directly impact cell adhesion, growth, and mineralization. The biological performance of the scaffolds was evaluated with MC3T3-E1 pre-osteoblastic cells through assays of cell adhesion, viability, proliferation, ALP activity, calcium secretion, and collagen production. This comparative approach provides insights into the potential of natural polysaccharides and laponite nanoclay for designing multifunctional scaffolds that recapitulate the organic and inorganic phases of native bone tissue.

## 2. Materials and Methods

### 2.1. Materials

Gelatin from bovine skin, polyvinyl alcohol (Mw 145,000 kg/mol), kappa-carrageenan, iota-carrageenan, glutaraldehyde, potassium chloride, L-ascorbic acid, β-glycerophosphate, and dexamethasone were purchased from Sigma-Aldrich (St. Louis, MO, USA). Laponite XLG was purchased from BYK Additives Ltd. (Widnes, UK). The PrestoBlue™ cell viability reagent was purchased from Invitrogen Life Technologies (Carlsbad, CA, USA). Trypsin/EDTA (0.25%) and phosphate-buffered saline (PBS) were purchased from Gibco ThermoFisher Scientific (Waltham, MA, USA). Paraformaldehyde (PFA), p-nitro-phenyl-phosphate (pNPP), Triton X-100, and Sirius Red 90 were purchased from Sigma-Aldrich (St. Louis, MO, USA). Penicillin/streptomycin was purchased from Gibco ThermoFisher Scientific (Waltham, MA, USA). Alpha-MEM cell culture medium and fetal bovine serum (FBS) were purchased from PAN-Biotech (Aidenbach, Germany). O-cresol phthalein complexone reagent kit was purchased from Biolabo (Maizy, France). Bradford reagent was purchased from AppliChem GmbH (Darmstadt, Germany).

### 2.2. Fabrication of the 3D Scaffolds

For the preparation of the 3D scaffolds, 1% *w*/*v* of iota- and kappa-carrageenan were employed, while keeping the PVA/gelatin concentration constant. Prior to mixing, all ingredients were sterilized under ultraviolet light. Initially, 3% *w*/*v* poly(vinyl alcohol) was dissolved in deionized water at 90 °C under continuous stirring for 4 h. After cooling the PVA solution to 50 °C, 3% *w*/*v* gelatin was added, and the mixture was stirred for an additional 2 h. Subsequently, the appropriate carrageenan powder was directly added to the PVA/gelatin solution, and the resulting blend with final concentrations of 3% *w*/*v* PVA, 3% *w*/*v* gelatin and 1% *w*/*v* KC or IC was stirred at a constant temperature of 50 °C for 4 h to ensure complete homogeneity.

For the samples containing Laponite XLG, the same procedure was followed, with the modification that different amounts of laponite were added to the cooled PVA solution prior to the addition of gelatin and carrageenan, to ensure complete dispersion of the clay. An initial attempt using 2% *w*/*v* laponite resulted in the formation of a highly viscous gel, which hindered its integration with the other components and led to visible aggregates. Therefore, two concentrations of laponite, 0.5% and 1% *w*/*v*, were used in preparations. Each mixture was stirred for 4 h before the addition of gelatin and carrageenan.

The final solutions were cast into 96- and 48-well plates and stored at −23 °C for 4 h, followed by lyophilization at −40 °C overnight to achieve physical crosslinking [15]. After the freeze-drying process, chemical crosslinking was carried out in two steps. The first step was performed using 0.25% *w*/*v* glutaraldehyde for 30 min. All samples were then rinsed twice with deionized water and subjected to a second crosslinking step using 0.2 M potassium chloride (KCl) for the same duration [10]. To ensure the removal of any unbound crosslinker residues, all scaffolds were thoroughly rinsed with ultrapure water. Table 1 summarizes the different scaffold types, including their compositions, concentrations, and crosslinking methods.

### 2.3. Physicochemical Characterization of the Scaffolds

#### 2.3.1. Fourier Transform Infrared Spectroscopy (FTIR)

FTIR analysis was performed on the different blend scaffold compositions, and their components including gelatin, PVA, kappa- and iota-carrageenan, and laponite. The infrared spectra were recorded in transmittance mode using a Nicolet 6700 (Thermo Scientific, Waltham, MA, USA) optical spectrometer over the range of 4000–400 cm^−1^, with 64 scans collected for each spectrum. The spectral data were graphically represented using Python 3.3 (Jupyter Notebook 7.5).

#### 2.3.2. X-Ray Diffraction Analysis (XRD)

The phase composition of pure substrates and scaffolds was investigated using a SmartLab SE X-ray diffractometer (Rigaku Co., Tokyo, Japan) equipped with Cu Kα radiation (λ = 1.5406 Å). Before measurement, the scaffolds were freeze-dried for 24 h to remove residual water and minimize background noise. All XRD measurements were carried out at room temperature using a five-axis goniometer with an in-plane arm, operating at 40 kV and 50 mA, with a step size of 0.01°/2θ. Data were collected over a 2θ range of 5–65°. The spectral data were graphically represented using Python (Jupyter Notebook).

#### 2.3.3. Surface Morphology and Elemental Composition of the Scaffolds

The surface morphology and microstructure of the scaffolds were examined by JSM-6390 LV scanning electron microscope (SEM) (JEOL Ltd., Tokyo, Japan) operated at an accelerating voltage of 20 kV. In brief, before imaging, freeze-dried scaffolds were sputter-coated with a 10 nm Au layer (Baltec SCD 050, Los Angeles, CA, USA) to enhance conductivity. The elemental analysis of the scaffolds was performed simultaneously using energy-dispersive X-ray spectroscopy (EDS) integrated within the SEM. The resulting X-ray spectra generated by the interaction of the electron beam with the sample were analyzed to identify and quantify the elemental composition of the scaffolds.

#### 2.3.4. Water Uptake Measurements

To quantify the swelling capacity of the scaffolds, after the crosslinking process, they were freeze-dried for 12 h, and their dry weight was measured (weight_dry_). Furthermore, the scaffolds were immersed in phosphate-buffered saline (PBS) at pH 7.4 and placed for 1 and 24 h at 37 °C in a CO_2_ Thermo Scientific™ incubator (Waltham, MA, USA). After incubation, the excess PBS was carefully removed by gently dabbing the samples with a paper towel, and their swollen weight was measured (weight_wet_). All weight measurements were conducted using a Kern ABT 220-5DNM high-precision analytical balance (Balingen-Frommern, Germany) with a readability of ±1 μg. All samples were analyzed in groups of three (*n* = 3). The swelling ratio was calculated following the formula:

(1) $$Swelling\;ratio\%=\frac{{weight}_{wet}-{weight}_{dry}}{{weight}_{dry}}\times 100\%$$

#### 2.3.5. In Vitro Degradation Testing

For the degradation study, the scaffolds were immersed in PBS (pH 7.4) and incubated at 37 °C. At selected time points (7, 14, 21, 28, and 40 days), the scaffolds were removed from the medium, gently dabbed with a paper towel to remove the excess of PBS, and then weighed to assess their degradation. Samples were analyzed in groups of five (*n* = 5). The degradation ratio was calculated using the formula:

(2) $$Degradation\;ratio\%=\frac{{weight}_{initial}-{weight}_{timepoint}}{{weight}_{initial}}\times 100\%$$

#### 2.3.6. Ion Release Study

To evaluate the release of inorganic ions from the scaffolds, acellular samples were immersed in 100 μL of alpha-MEM medium and incubated at 37 °C for 1, 7, 14, and 21 days. The supernatants were collected at each time point, and analyzed using the O-cresol phthalein complexone (CPC) dye. Although the CPC assay is primarily designed to quantify Ca^2+^, it can also form colored complexes with other divalent cations, including Ba^2+^, Mg^2+^, Sr^2+^, Pb^2+^, and Zn^2+^ [31]. Because laponite releases Mg^2+^ upon dissolution, the absorbance measured in CPC assays reflected the presence of Mg^2+^ ions rather than calcium. For each sample, 10 μL of the supernatant were mixed with 100 μL of calcium buffer and 100 μL of the CPC dye [10], transferred into a 96-well plate, and the absorbance was recorded at 570 nm using a spectrophotometer. Samples were analyzed in quadruplicate, and ion concentrations are reported after subtraction of the baseline values measured in alpha-MEM medium alone.

#### 2.3.7. Density and Porosity

The apparent density and porosity of the scaffolds were measured using the liquid displacement technique [32,33]. In this method, ethanol was selected as the wetting agent since it easily penetrates the scaffolds’ pores without causing shrinkage, swelling, or dissolution at room temperature. Briefly, a known mass of freeze-dried scaffolds (W) was immersed in a graduated cylinder containing a known volume of ethanol (V_1_) and ultrasonically stirred until their pores were completely filled with ethanol, no air bubbles were observed, and the specimens were fully submerged. The total volume was then recorded as V_2_. Subsequently, the scaffolds were withdrawn from ethanol, and the remaining ethanol volume was designated as V_3_. The average values of five measurements were taken to validate the porosity and density of the specimens. The density and porosity of the scaffolds were calculated by the following equations:

(3) $$Density=\frac{W}{V_{2}-V_{3}}$$

(4) $$Porosity=\frac{V_{1}-V_{3}}{V_{2}-V_{3}}$$

#### 2.3.8. Mechanical Characterization of the Scaffolds

The effect of carrageenan and laponite incorporation into PVA/gelatin on the mechanical properties of the hydrogels was evaluated by uniaxial compression tests. Testing was conducted using a UniVert mechanical test system (CellScale, Waterloo, ON, Canada) with a maximum load of 50 N. The Young’s modulus was measured at the linear regime of a strain range of 40–60%, with a deformation speed of 5 mm/s, using six replicates for each scaffold composition (*n* = 6). Young’s modulus values of the scaffolds were determined as the slope in the linear elastic deformation regime of the stress–strain graph according to the formula:

(5) $$Young’s\;modulus\;E=\frac{F\cdot L}{A\cdot \Delta L}\;,$$

where F stands for the perpendicular force applied to the surface area A of the scaffold, ΔL is the change in scaffold height after force application, and L is the initial height of the samples.

### 2.4. In Vitro Biological Characterization of the Scaffolds

#### 2.4.1. Cell Culture and Cell Viability Evaluation

To estimate the biological performance of the scaffolds, the MC3T3-E1 mouse calvaria osteoblast precursor cell line (ACC-210, Leibniz Institute DSMZ, Germany) was used as the model for in vitro assays. Cells of passage 19 were cultured in alpha-MEM medium supplemented with 10% fetal bovine serum (FBS) and 1% penicillin/streptomycin in an incubator at 37 °C and 5% CO_2_. Before the cell seeding, all scaffolds were sterilized by UV irradiation for 15 min on each side. A suspension of 5 × 10^3^ cells in 10 μL of complete medium was carefully pipetted onto each scaffold, followed by incubation for 1 h to allow cell attachment. Subsequently, culture medium (160 μL per well) was added to each scaffold. The medium was refreshed every two to three days.

The viability of the MC3T3-E1 pre-osteoblast cells was determined using the PrestoBlue™ cell viability assay. At each time point (3, 7, and 14 days), 10 μL of PrestoBlue™ reagent diluted 1:10 in fresh α-MEM™ medium was added to each well and incubated at 37 °C and 5% CO_2_ for 1 h. After that, 100 μL of the supernatant of each sample was transferred to a 96-well plate, and the absorbance was measured at 570 and 600 nm using a spectrophotometer. Samples were analyzed in quintuplicate.

#### 2.4.2. SEM Imaging

The cell-seeded scaffolds intended for SEM visualization were incubated at 37 °C with 5% CO_2_. On day 1, the cell-loaded scaffolds were rinsed with PBS, fixed with 4% *v*/*v* paraformaldehyde for 25 min, and placed at −23 °C. After the stabilization period, the samples were washed again with PBS to remove any remaining fixative, and dehydration occurred through 10 min washes with gradually increasing ethanol concentrations (30, 50, 70, 90, and 100% *v*/*v*). Afterwards, the samples were sputter-coated with gold for 90 s, following a similar procedure to that applied for the scaffolds without cells.

#### 2.4.3. Measurement of the Alkaline Phosphatase Activity 

Alkaline phosphatase (ALP) is a key enzyme expressed in mineralized tissues, particularly during bone formation, where it detaches phosphate groups and facilitates the production of hydroxyapatite crystals. These crystals are a fundamental component of the extracellular matrix, as they accumulate between collagen fibrils and contribute to the mineralization of bone tissue [34]. The ALP activity is an early-stage indicator of osteogenesis; therefore, 40 × 10^3^ pre-osteoblastic cells were seeded onto the various scaffold types, and cultured for 3, 7, and 14 days. To induce osteogenic differentiation, the cell-loaded scaffolds were cultured in cell culture medium supplemented with the osteogenic cocktail that includes 50 μg/mL l-ascorbic acid, 10 mM β-glycerophosphate, and 10 nM dexamethasone.

Based on a previously established protocol [35], the cell-loaded scaffolds were washed three times with PBS, covered with 100 μL of cell lysis buffer containing 0.1% Triton X-100 and 50 mM Tris-HCl (pH 10.5), and then subjected to two freezing/thawing cycles between −23 °C and room temperature. After that, 100 μL of the suspension from each well was mixed with 100 μL of a 2 mg/mL p-nitro-phenyl-phosphate (pNPP) solution diluted in 50 mM Tris-HCl and 2 mM MgCl_2_ buffer, and the total mixtures were incubated for 1 h at 37 °C. Following incubation, 100 μL of each sample were transferred to a 96-well plate and the color change was measured at 405 nm using a BioTek Synergy HTX Multi-Mode Micro-plate Reader (Bad Friedrichshall, Germany). The enzymatic activity was expressed as nmol pNPP/min, and normalized to the total cellular protein content, which was determined using the Bradford method for protein concentration measurement. Samples were analyzed in five replicates.

#### 2.4.4. Assessment of Calcium Concentration Levels

Calcium production is associated with the formation of hydroxyapatite, and its deposition is considered a late marker of osteogenesis, indicating the formation of the ECM. To determine calcium secretion, the O-cresol phthalein complexone method was employed. CPC reacts in alkaline environment with calcium to form a dark-red complex, and the absorbance of this is proportional to the calcium concentration. Supernatants from cell-seeded scaffolds containing 40 × 10^3^ MC3T3-E1 cells were collected every three days up to day 21. For analysis, 10 μL of culture medium from each sample was mixed with 100 μL of calcium buffer and 100 μL of calcium dye [10]. The mixtures were transferred to 96-well plates, and absorbance was measured at 570 nm using a spectrophotometer. Values of cell-free scaffolds have been subtracted as blank. Samples were analyzed in quadruplicate.

#### 2.4.5. Determination of Secreted Collagen

To measure collagen production by cultured cells on the scaffolds, the Sirius Red Dye assay was used. The anionic Sirius Red dye contains negatively charged sulfonic acid groups that ionically bind to the positively charged amino groups of the various collagen types produced by the cells [36]. A total of 40 × 10^3^ pre-osteoblastic cells were seeded onto the different scaffolds. At each time point (7, 14, and 21 days), 25 μL of each supernatant was diluted in 75 μL of dH_2_O in a 2 mL Eppendorf tube. Then, 1 mL of 0.1% *w*/*v* Sirius Red Dye solution in 0.5 M acetic acid was added, and the samples were incubated at room temperature for 45 min. After the time had passed, the samples were centrifuged for 20 min at 15,000× *g*. The supernatant was poured off and replaced with 1 mL of fresh acetic acid. This step was repeated to remove any residual non-bound dye until the supernatant was transparent. To release the bound dye from the collagen pellet, 1 mL of 0.5 M NaOH was added to each tube, vortexed, and 200 μL of the resulting solution was transferred to a 96-well plate to measure its absorbance at 530 nm. A standard curve was prepared to correlate absorbance values with collagen concentration (μg/mL). Samples were analyzed in groups of six (*n* = 6).

### 2.5. Statistical Analysis

Statistical analysis was performed using GraphPad Prism 8 software to evaluate the significance of the differences between various scaffold compositions and the PVA/GEL control (*), between KC and IC scaffolds (#), and between LAP0.5 and LAP1 (§). A two-way ANOVA Dunnett’s multi-comparison test was performed for degradation, ion release, cell viability, ALP activity, calcium production, and collagen secretion. Compression, porosity, and density were analyzed with one-way ANOVA Dunnett’s multi-comparison test, while swelling capacity was measured through two-way ANOVA Sidak’s multi-comparison test. Data are expressed as means ± standard deviation (SD), with *p* < 0.05 considered statistically significant.

## 3. Results

### 3.1. FTIR Characterization

**Figure 1:**
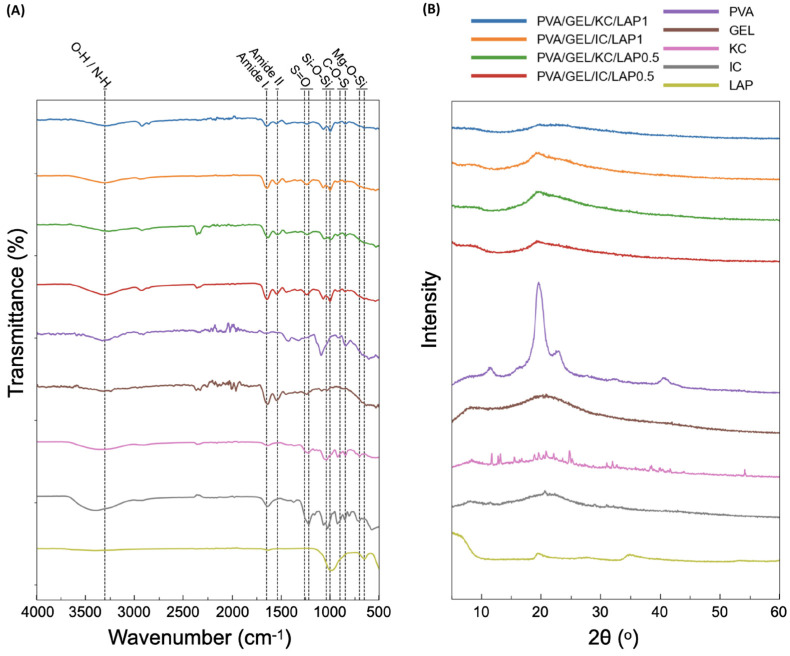
(**A**) Fourier-transform infrared spectra and (**B**) X-ray diffraction patterns of the individual components in powder form (PVA, GEL, KC, IC, and LAP), alongside the compositions PVA/GEL/KC/LAP0.5, PVA/GEL/IC/LAP0.5, PVA/GEL/KC/LAP1, and PVA/GEL/IC/LAP1.

The FTIR spectra (Figure 1B) of the individual ingredients confirmed the characteristic absorption bands of each component. PVA displayed prominent peaks at approximately 3300 cm^−1^ corresponding to O-H stretching vibrations, along with peaks around 2940 cm^−1^ (C-H stretching) and 1090 cm^−1^ (C-O stretching of the polymer backbone). Pure gelatin exhibited a stretch at 3200–3500 cm^−1^ because of the presence of N-H bonds as well as O-H bonds that are found in the same region. Additionally, there are amide bands at 1650 cm^−1^ (amide I, C=O stretching), 1540 cm^−1^ (amide II, N-H bending), and 1240 cm^−1^ (amide III), indicative of its proteinaceous structure. Both kappa-carrageenan (KC) and iota-carrageenan (IC) showed characteristic sulfate ester peaks between 1220 and 1260 cm^−1^ (S=O stretching) and 900–850 cm^−1^ (C-O-S vibration), assigned to 3,6-anhydrogalactose [37] and confirming their sulfated polysaccharide nature [38]. Moreover, their stretch at 3500–3000 cm^−1^ can be attributed to the O-H groups that carrageenan possesses [39]. Laponite presented intense silicate vibrations at 970–1040 cm^−1^ (Si-O-Si/Si-O) together with weaker bands at 650–700 cm^−1^ (Mg-O-Si bending), consistent with the silicate framework of the nanoclay [40,41]. These reference spectra establish the band positions used to track component incorporation in the composites.

All composite formulations retained the characteristic signatures of their constituents, confirming successful component integration into the polymeric matrices. The broad O-H stretching band between 3200 and 3500 cm^−1^ was observed in all formulations, became wider and slightly shifted, consistent with extensive hydrogen bonding among the polymers and LAP. Amide I and II peaks from gelatin remained visible at 1650 and 1540 cm^−1^, confirming protein retention within the blends. The sulfate ester peaks from carrageenans (S=O, at 1260–1220 cm^−1^ and 900–845 cm^−1^ regions) were preserved in the corresponding KC- and IC-based scaffolds, although with reduced intensity, likely due to overlapping with LAP and PVA contributions. Moreover, the characteristic Si-O vibrations (1040–1000 cm^−1^) became more pronounced in LAP-containing scaffolds, with their intensity increasing proportionally with higher laponite loading, reflecting the successful integration of the nanoclay into the polymer network. Overall, the FTIR data validate the chemical composition of the developed scaffolds and confirm molecular-level interactions between the polymers and laponite, which may underpin the enhanced structural and functional properties observed in subsequent analyses.

### 3.2. X-Ray Diffraction Analysis

XRD measurements were performed to assess the impact of laponite nanoplatelets on the crystallinity of the PVA/GEL backbone. The XRD spectra (Figure 1B) of the individual components confirmed their crystalline or amorphous features. PVA displayed a sharp semicrystalline reflection at 2θ of 19.5° [42], and gelatin showed a broad amorphous halo centered near 20°, which is typical of its denatured collagen backbone [43]. Kappa-carrageenan exhibited multiple crystalline reflections between 10 and 30°, indicative of an ordered polysaccharide structure [44], whereas iota-carrageenan presented a broader halo around 22°, consistent with a partially amorphous structure [45]. Finally, laponite was characterized by low-angle basal reflections (below 10°), together with silicate peaks at 19.8° and 35° characteristic of its layered silicate lattice [46,47].

In the composite scaffolds containing laponite, the diffraction patterns preserved the main features of the polymeric matrices, but they became slightly broader and less intense, suggesting changes in chain packing upon nanoclay addition. All nanocomposites were dominated by a broad halo centered at 2θ of 20–22°, consistent with an amorphous polymeric matrix [48]. Silicate-associated features were retained but broadened, and their intensity increases visibly with higher nanoclay loading, from 0.5 to 1% *w*/*v*, especially in the region between 19 and 22, the LAP (001) reflection at 6–8° is strongly attenuated, indicating extensive layer delamination/intercalation within the matrix rather than persistence of stacked tactoids. No new crystalline phases were detected upon LAP addition, suggesting that LAP incorporation does not induce crystallization of the polymer network but is dispersed at the nanoscale. Overall, the XRD patterns indicate effective integration and high dispersion of laponite within the PVA/GEL-carrageenan scaffolds while preserving a largely amorphous matrix.

### 3.3. Scaffolds’ Morphology

**Figure 2:**
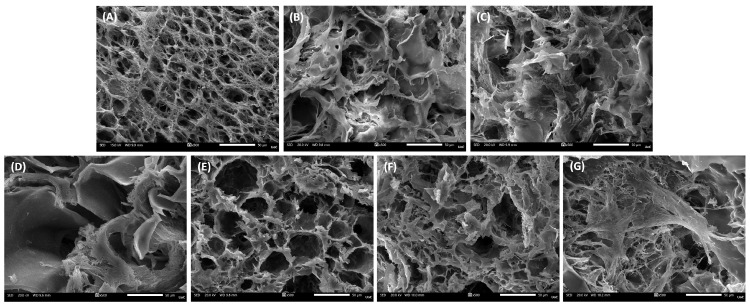
Representative SEM images of cell-free scaffold cross-sections at a magnification of 500× (scale bar represents 50 μm). (**A**) PVA/GEL, (**B**) PVA/GEL/KC, (**C**) PVA/GEL/IC, (**D**) PVA/GEL/KC/LAP0.5, (**E**) PVA/GEL/IC/LAP0.5, (**F**) PVA/GEL/KC/LAP1, (**G**) PVA/GEL/IC/LAP1.

The microstructural features of the scaffolds were assessed through SEM cross-sections (Figure 2A–G). All formulations displayed a porous and interconnected architecture, which is crucial for facilitating nutrient diffusion and potential cell infiltration. The PVA/GEL control scaffold (Figure 2A) exhibited a uniform network, whereas carrageenan incorporation (Figure 2B,C) resulted in a visibly more open and interconnected morphology, with loosely arranged pore walls. The addition of laponite (Figure 2D–G), preserved overall porosity but introduced a denser morphology, particularly at higher concentrations, indicating the reinforcing effect of the nanoclay on the scaffold matrix.

Complementary EDS analysis provided semi-quantitative confirmation of the elemental composition of the scaffolds (Table 2). Carbon (C) and oxygen (O) were predominant in all groups, ranging between 49 and 58% and 39–42%, respectively, reflecting the organic polymeric backbone. Weak, but consistent, signals of chlorine (Cl, 1–2%) and potassium (K, 1–3%) were detected, possibly attributed to residuals of the ionic crosslinker KCl, and sulfur (S, <1%) due to the sulfate groups of carrageenans. Upon laponite inclusion, additional peaks corresponding to magnesium (Mg, up to 2%), silicon (Si, up to 4%) were additionally observed, with their relative mass percentage increasing at higher LAP content. These outcomes corroborate the incorporation of LAP into the polymeric matrices.

### 3.4. Structural and Mechanical Properties

**Figure 3:**
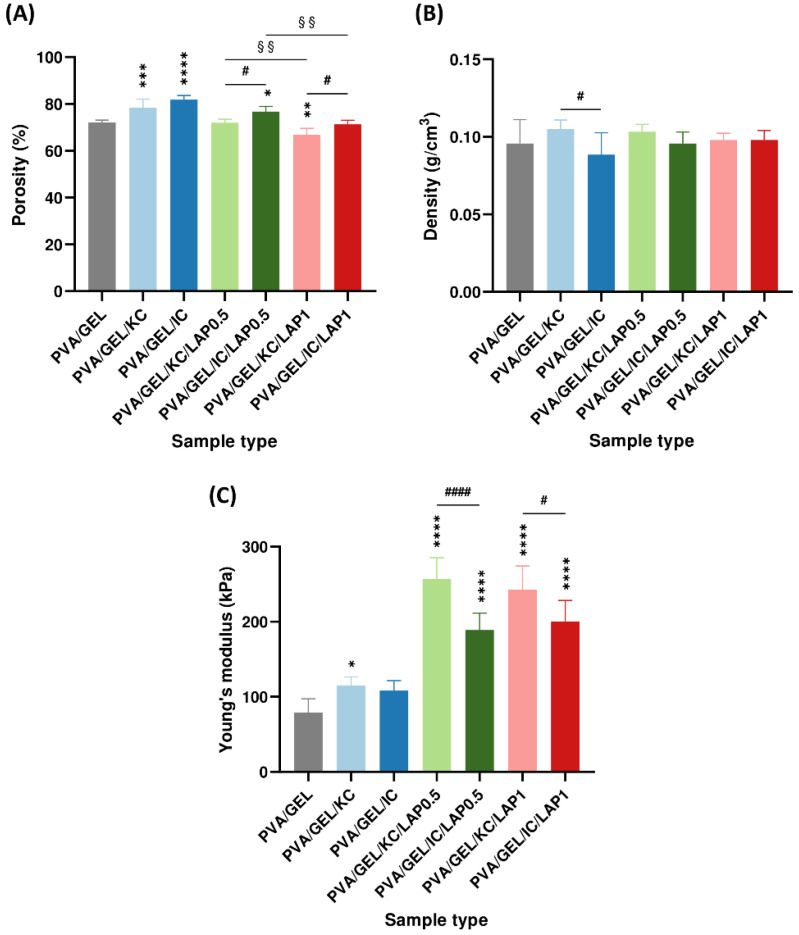
(**A**) Porosity (%), (**B**) density (g/cm^3^), and (**C**) Young’s modulus (kPa) of the scaffolds. Young’s modulus was measured at the linear regime of 40–60% strain and 5 mm/sec compression speed. Data are presented as mean ± SD. Statistical significance relative to the PVA/GEL control scaffold is indicated as * *p* < 0.05, ** *p* < 0.01, *** *p* < 0.001, **** *p* < 0.0001; differences between KC and IC are shown as # *p* < 0.05, #### *p* < 0.0001; and between LAP0.5 and LAP1 are §§ *p* < 0.01.

As shown in Figure 3A, the porosity of the scaffolds exhibited clear compositional dependence. The PVA/GEL/IC exhibited the highest values of 82%, followed by PVA/GEL/KC of 78%, both significantly higher in comparison to the PVA/GEL control at 72%. The addition of laponite did not further raise porosity; instead, it partially offset the carrageenan-driven increase, resulting in values comparable or slightly below the control. However, in Figure 3B, the apparent density of the scaffolds remained relatively unchanged across all formulations, ranging between 0.09 and 0.11 g/cm^3^, without any statistically significant differences observed, suggesting that the porosity changes did not substantially alter the bulk density of the scaffolds.

Mechanical testing (Figure 3C) revealed clear distinctions between the scaffold types. PVA/GEL/KC and PVA/GEL/IC scaffolds showed only a modest enhancement in Young’s modulus relative to the control, with a value slightly above 100 kPa. By contrast, the LAP-containing scaffolds exhibited substantial reinforcement, with modulus values consistently exceeding 190 kPa and in some cases approaching 260 kPa. In particular, the polymer-nanoclay interactions acted as a reinforcing agent, markedly strengthening the polymer matrix, while carrageenan contributed to maintaining structural openness. This combination yielded scaffolds that paired high permeability with mechanical robustness, sustaining porosity close to the control while more than doubling the modulus.

### 3.5. Swelling and Degradation Analysis

**Figure 4:**
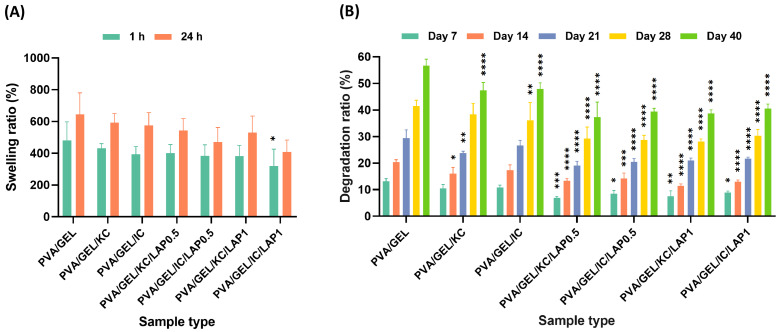
(**A**) Swelling ratio values of the scaffolds after 1 and 24 h of immersion in PBS, and (**B**) degradation ratio values of the various scaffold compositions after 7, 14, 21, 28, and 40 days of immersion in PBS. Statistical analysis was considered at * *p* < 0.05, ** *p* < 0.01, *** *p* < 0.001, **** *p* < 0.0001 when compared to the PVA/GEL control scaffold. Statistical analysis between KC and IC, as well as between LAP0.5 and LAP1 groups, indicated non-significant differences in swelling and degradation rate.

The swelling capacity of the scaffolds was evaluated after 1 and 24 h of immersion in PBS (Figure 4A). Even within the first hour, all compositions surpassed their dry weight, reaching approximately 400% and further increasing to nearly 520% at 24 h, reflecting the progressive uptake of PBS over time. Specifically, after 24 h, the PVA/GEL/KC and PVA/GEL/IC scaffolds demonstrated the highest swelling ratios (593 ± 47% and 574 ± 67%, respectively). Nonetheless, the inclusion of laponite slightly reduced swelling, particularly in the PVA/GEL/IC/LAP1 scaffold, which showed the lowest value (408 ± 62%) compared to the PVA/GEL control. These results suggest that KC and IC enhance water absorption, whereas laponite restricts fluid uptake by reinforcing the scaffold network.

The degradation behavior of the scaffolds was assessed over 7, 14, 21, 28, and 40 days of immersion in PBS at 37 °C (Figure 4B). All samples displayed a gradual mass loss over time, with the PVA/GEL control scaffolds degrading at a higher rate throughout the study, reaching nearly 57% weight loss by day 40. While the incorporation of carrageenans moderately reduced the degradation ratio, a clear correlation with the swelling profiles was evident. The PVA/GEL/KC and PVA/GEL/IC scaffolds, which exhibited higher swelling capacities, degraded faster (53 ± 2% mass loss for both), consistent with their more open and hydrated network structures. Conversely, the presence of laponite markedly improved long-term stability by reinforcing the polymer matrix, with all four laponite-containing scaffolds maintaining more than 60% of their initial weight after 40 days.

### 3.6. Ion Release

**Figure 5:**
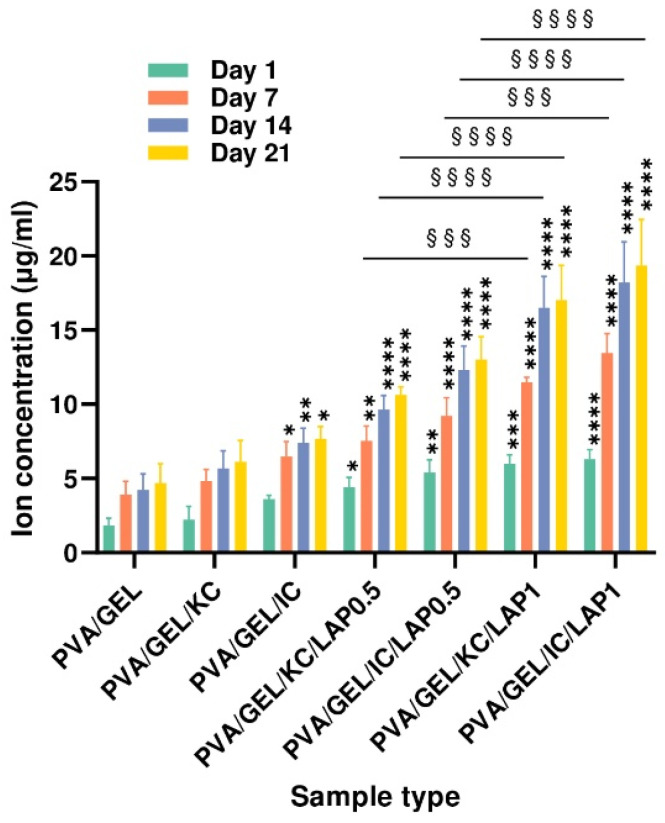
Cumulative ion concentration released from acellular scaffolds on days 7, 14, and 21. Data represent the mean ± SD (*n* = 4) (* *p* < 0.05, ** *p* < 0.01, *** *p* < 0.001, **** *p* < 0.0001 compared to the PVA/gelatin control at the corresponding timepoint; and §§§ *p* < 0.001, §§§§ *p* < 0.0001 are between LAP0.5 and LAP1; statistical analysis between KC and IC groups indicated non-significant differences).

The concentration of released ions increased progressively over the 21-day incubation period for all scaffold compositions (Figure 5). Samples containing laponite exhibited the highest concentrations at each time point up to 21 days, confirming progressive Mg^2+^ release during incubation. The low values observed in the laponite-free scaffolds, namely the PVA/GEL as well as the KC or IC scaffolds, which are between 2 and 7 μg/mL, may be attributed to the ions present in the cell culture medium. In contrast, all laponite-containing scaffolds showed a pronounced, composition-dependent enhancement in ion release relative to their counterparts without laponite. For both kappa- and iota-carrageenan formulations, increasing the laponite content from 0.5% to 1% produced a stepwise rise in ion concentration at each time point. After 21 days, the PVA/GEL/KC/LAP1 and PVA/GEL/IC/LAP1 scaffolds released the highest ion levels, reaching approximately 20–23 μg/mL. In contrast, the corresponding 0.5% LAP composites displayed intermediate release values of 12–16 μg/mL, indicating that the ion release from the scaffolds scales with the amount of incorporated laponite.

### 3.7. Adhesion, Morphology, Viability, and Proliferation of Pre-Osteoblasts Inside the Scaffolds

**Figure 6:**
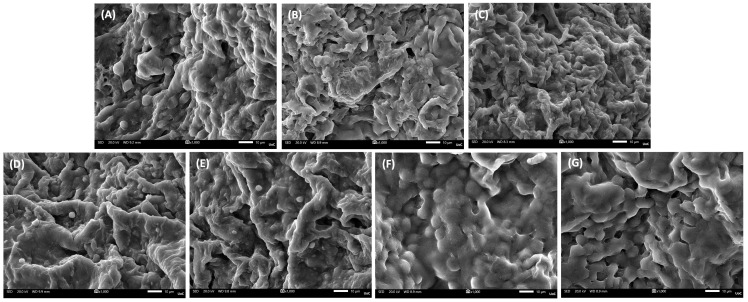
Representative SEM images of cell adhesion on the various scaffold compositions on day 1 (magnification 1000×; scale bar 10 µm). (**A**) PVA/GEL, (**B**) PVA/GEL/KC, (**C**) PVA/GEL/IC, (**D**) PVA/GEL/KC/LAP0.5, (**E**) PVA/GEL/IC/LAP0.5, (**F**) PVA/GEL/KC/LAP1, (**G**) PVA/GEL/IC/LAP1.

SEM imaging of cell-loaded scaffolds on day 1 (Figure 6A–G) revealed robust early adhesion across all formulations, with differences in the distribution of cells adhering along pore walls and bridging microcavities. Cells seeded on PVA/GEL control (Figure 6A) exhibited limited attachment and spreading, with some round cells remaining only partially adhered. In contrast, carrageenan-containing scaffolds (KC and IC; Figure 6B,C) displayed improved cell-scaffold interactions, with more uniform cell coverage across the scaffold surface. LAP-bearing scaffolds, both LAP0.5 (Figure 6D,E) and LAP1 scaffolds (Figure 6F,G), presented denser cell layers and elongated morphologies.

**Figure 7:**
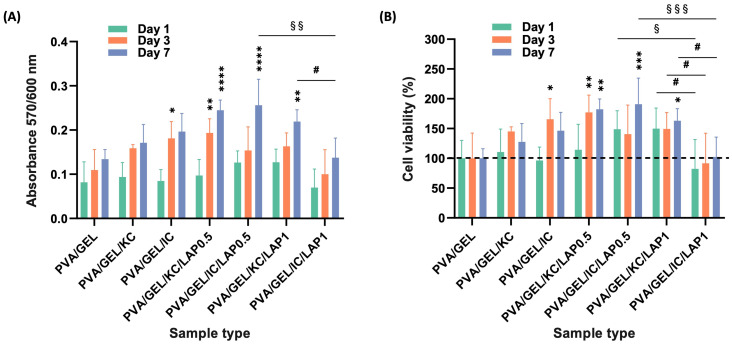
(**A**) Cell viability and proliferation assessment expressed as absorbance and (**B**) as % viability on days 1, 3, and 7 (the dashed line represents the 100% level). Data are shown as mean ± SD of *n* = 5 (* *p* < 0.05, ** *p* < 0.01, *** *p* < 0.001, **** *p* < 0.0001 compared to the PVA/gelatin control; # *p* < 0.05 shows differences between KC and IC; and § *p* < 0.05, §§ *p* < 0.01, §§§ *p* < 0.001 indicates differences between LAP0.5 and LAP1 at the corresponding timepoint).

The metabolic activity of living pre-osteoblastic cells per scaffold was quantified on days 1, 3, and 7 and expressed in absorbance values (Figure 7A). All scaffold types featured a time-dependent increase in absorbance, with LAP-containing formulations depicting statistically higher activity than the PVA/GEL control. Notably, PVA/GEL/KC/LAP0.5 and PVA/GEL/KC/LAP1 reached the highest values on day 7, indicating enhanced cell proliferation. Carrageenan alone induced only modest increases.

Cell viability % normalized to the PVA/GEL control (set at 100%) (Figure 7B) confirmed sustained survival on all scaffold conditions, with viability consistently above 100% throughout the 7-day culture period, indicating the absence of cytotoxicity. However, a decrease in viability was observed on day 7 for the PVA/GEL/KC and PVA/GEL/IC scaffolds, dropping to 128 ± 27% and 146 ± 26%, respectively, after reaching peak values of 145 ± 7% and 166 ± 30% on day 3. Contrarily, the LAP0.5 formulations (with both KC and IC) again promoted the greatest increase, reaching nearly 200% viability by day 7, whereas 1 wt% LAP scaffolds remained closer to baseline and showed reduced viability, suggesting a concentration-dependent effect of LAP on cell response. Notably, on day 7, PVA/GEL/KC/LAP1 scaffolds showed a significantly higher viability than PVA/GEL/IC/LAP1, indicating that the type of carrageenan modulates the cellular response to high laponite concentration. Altogether, these data demonstrate that while all formulations support cell attachment and biocompatibility, scaffold composition strongly influences early cell adhesion and proliferation. The incorporation of laponite synergistically with either carrageenan created the most favorable microenvironment for early proliferation.

### 3.8. Osteogenic Differentiation

**Figure 8:**
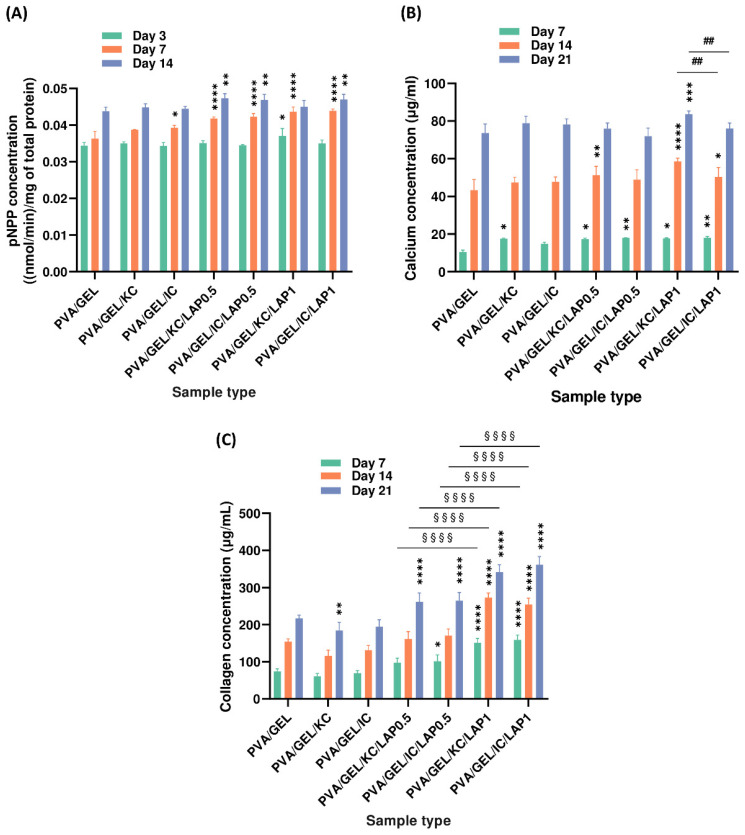
Markers of osteogenic activity of pre-osteoblastic cells cultured on scaffolds. (**A**) ALP activity on days 3, 7, and 14; (**B**) calcium secretion and (**C**) total collagen secretion on days 7, 14, and 21. Data are presented as mean ± SD (* *p* < 0.05, ** *p* < 0.01, *** *p* < 0.001, **** *p* < 0.0001 compared to the PVA/gelatin control; ## *p* < 0.01 shows differences between KC and IC; and §§§§ *p* < 0.0001 are between LAP0.5 and LAP1, at the corresponding timepoint).

Alkaline phosphatase (ALP) enzymatic activity, an early marker of osteogenic differentiation, was quantified on days 3, 7, and 14 (Figure 8A). All scaffold compositions supported ALP expression, with activities increasing steadily over time. LAP-containing composites, particularly PVA/GEL/KC/LAP0.5, PVA/GEL/IC/LAP0.5, and PVA/GEL/IC/LAP1, evidenced statistically higher ALP activity on day 14 compared to the PVA/GEL control, suggesting that LAP incorporation enhances early osteogenic differentiation. Conversely, carrageenan-containing scaffolds (PVA/GEL/KC and PVA/GEL/IC) yielded only modest gains over the control.

Calcium secretion, reflecting matrix mineralization, was determined on days 7, 14, and 21 (Figure 8B). A time-dependent increase in calcium accumulation was observed for all groups. By day 14, scaffolds containing LAP displayed a clear enhancement in mineralization, particularly PVA/GEL/KC/LAP0.5 (51 ± 4 µg/mL), PVA/GEL/KC/LAP1 (59 ± 1 µg/mL), and PVA/GEL/IC/LAP1 (50 ± 4 µg/mL), which were significantly higher than both carrageenan-containing scaffolds (47 ± 2 µg/mL) and the PVA/GEL control (43 ± 5 µg/mL). This trend was further amplified on day 21, where PVA/GEL/KC/LAP1 reached the highest calcium concentrations (84 ± 1 µg/mL), surpassing all other scaffold compositions. These findings indicate that laponite acts as a potent promoter of mineral deposition within the composite network.

The total collagen production (Figure 8C), another marker of ECM maturation, was evaluated over 21 days. A progressive trend was observed across all groups. However, the carrageenan-containing scaffolds (PVA/GEL/KC and PVA/GEL/IC) consistently showed reduced secretion than the PVA/GEL control, reaching 65 ± 6 µg/mL on day 7, 124 ± 12 µg/mL on day 14, and 190 ± 18 µg/mL on day 21. Nevertheless, the laponite samples steadily outperformed the control, with concentrations exceeding 264 ± 20 µg/mL (PVA/GEL/KC/LAP0.5 and PVA/GEL/IC/LAP0.5) and 352 ± 18 µg/mL (PVA/GEL/KC/LAP1 and PVA/GEL/IC/LAP1) by day 21. As this effect was evident for both laponite concentrations (0.5 and 1 wt%) and with either carrageenan type, it indicates robust promotion of extracellular matrix synthesis.

## 4. Discussion

Tissue engineering aims to develop biomaterial-based scaffolds that provide cell-permissive microenvironments while ensuring sufficient structural support for tissue formation. Natural polymers offer excellent bioactivity, while synthetic polymers and inorganic additives contribute structurally to integrity and mechanical reinforcement. However, the limited mechanical resilience of natural biomaterials often necessitates physical/chemical crosslinking strategies, as well as compositional modifications, to optimize their performance for targeted biomedical applications. In this context, the present study focused on the development of PVA/GEL-based scaffolds reinforced with kappa- and iota-carrageenan and further functionalized with laponite, aiming to fabricate composite scaffolds with favorable physicochemical, mechanical, and biological properties suitable for bone tissue engineering. To enhance stability, a dual crosslinking strategy was applied, using a low concentration of glutaraldehyde to covalently link free amino groups of gelatin with hydroxyl groups in PVA and carrageenan, thereby strengthening the polymeric network [10,14]. In contrast, potassium chloride was used to ionically crosslink carrageenan, enhancing its gelling capacity and further stabilizing the porous architecture [24,49,50].

The chemical composition and successful incorporation of each constituent were confirmed by FTIR analysis, which revealed the characteristic absorption bands of PVA, gelatin, carrageenans, and laponite within the composite spectra. Gelatin’s amide I/II peaks (1650/1540 cm^−1^) and carrageenan’s sulfate signatures (1220–1260 cm^−1^; 900–850 cm^−1^) remained visible, indicating preservation of the biopolymer backbones. In LAP-containing scaffolds, broadening and slight shifting of the O-H stretching band indicated an extensive hydrogen-bonding network, while the Si-O vibrations increased proportionally with LAP content, confirming its stable integration into the polymer network. The absence of new diagnostic bands supports reinforcement dominated by physical interactions rather than new covalent bond formation.

Complementary XRD analysis further corroborated these findings, showing broad halos centered at 2θ of 20–22° characteristic of an amorphous polymer matrix. The attenuation of the basal LAP reflection at 6–8° suggested nanoscale dispersion of laponite within the polymeric network. Moreover, the increased intensity of silicate-associated features with higher LAP loading at 20° confirmed the presence of well-dispersed silicate domains without the formation of new crystalline phases. Together, these results indicate the effectiveness of polymer-nanoclay interactions and the homogeneous distribution of laponite within the scaffolds [28].

At the structural level, the addition of carrageenan increased pore openness without altering bulk density, whereas LAP preserved interconnectivity but densified the pore walls. As the laponite concentration increased, porosity was progressively reduced, producing a more compact and uniform pore architecture [51,52]. Nevertheless, the developed scaffolds exhibited a porosity of approximately 75%, which falls within the reported range for trabecular bone (50–90%) [53], and an apparent density maintained between 0.09 and 0.11 g/cm^3^, exhibiting their suitability for bone tissue engineering. Statistical comparisons revealed that porosity was significantly higher in IC-based scaffolds than in KC-based scaffolds. This result aligns with the higher sulfation degree of IC, which introduces more negatively charged sulfate groups, enhances electrostatic repulsion along the polysaccharide chains, increases water retention, and leads to a more hydrated and open polymer network [54]. Moreover, the successful integration of laponite into the scaffold matrix is further confirmed by Mg and Si elemental peaks detected by EDS, consistent with the incorporation of the nanoclay [55]. Given the highly porous nature of the scaffolds, the reported elemental values should be regarded as indicative of elemental presence and relative trends rather than exact stoichiometric ratios.

The obtained stress–strain curves in compression mode provided insight into the scaffolds’ load-bearing behavior. Carrageenan-containing scaffolds indicated modest improvements in compressive properties, whereas LAP incorporation led to a marked reinforcement of the matrix. The stress–strain response displayed an initial linear elastic stage, a plateau characterized by reduced stiffness due to progressive collapse of the pore walls, and a densification phase at strains above approximately 60%, where strain hardening occurred as the pores became fully compacted. Similar mechanical behavior has been reported in other gelatin-based soft tissue scaffolds containing carrageenan and chitosan [56], as well as in collagen-glycosaminoglycan scaffolds for bone tissue engineering, in which the transition from the elastic to the plateau and densification regions reflects the gradual deformation and compaction of the porous network [57]. Young’s modulus increased with laponite incorporation, exceeding 190 kPa, which remain below those of native cancellous bone reported at 50–500 MPa [58], but clearly indicate the reinforcing effect of laponite [59]. Notably, the modulus was significantly higher in KC-based scaffolds compared to IC-based ones. This behavior can be explained by the lower sulfation degree of KC, which reduces electrostatic repulsion between polymer chains and favors K^+^-mediated double-helix aggregation, resulting in a denser network with stronger intermolecular junctions and, consequently, a mechanically more resilient network [54,60]. This enhancement can be attributed to polymer-nanoclay interactions, which restrict polymer chain mobility and strengthen the composite matrix [59].

The swelling behavior of scaffolds is a critical parameter in tissue engineering, as it directly affects nutrient diffusion, waste removal, and the maintenance of a hydrated microenvironment, necessary for cell survival and proliferation. Scaffolds with higher fluid uptake can better emulate the native extracellular matrix, supporting matrix remodeling and tissue ingrowth, while excessive swelling may compromise structural integrity. Therefore, the swelling and degradation characteristics of the fabricated scaffolds were evaluated to assess their suitability for bone tissue regeneration. Hydration studies indicated that carrageenan-containing scaffolds exhibited the highest swelling ratios, reaching nearly 600% of their dry weight, in agreement with previous findings [4,56], while laponite reduced fluid uptake, which is in line with other reports demonstrating significant reduction in water uptake in the respective composite [16]. This inverse relationship between swelling and laponite concentration suggests that the nanoclay acts as a physical crosslinker, tightening the polymer network and restricting water diffusion. This reduction can be attributed to the inorganic nature of laponite, which forms strong physical interactions with polymer chains, limiting their mobility and decreasing the free volume available for water penetration [61]. Additionally, this behavior can be attributed to the hydrophilic and polyanionic nature of carrageenan, whose sulfated chains closely resemble glycosaminoglycans (GAGs) [62], promoting strong water uptake and matrix hydration [7,10]. In parallel, degradation assays revealed that higher swelling correlated with faster mass loss, with KC and IC scaffolds degrading more rapidly due to their highly hydrated and open structures. Conversely, the reduction in swelling upon laponite addition led to long-term stability, with over 60% of the initial mass retained after 40 days [56]. Collectively, these trends indicate that carrageenan and laponite act complementarily to balance permeability and mechanical stability in PVA/GEL-based scaffolds for bone tissue engineering. This balance between hydration and stability is crucial, as it determines the scaffold’s microenvironmental properties, affecting nutrient transport, matrix remodeling, and the maintenance of structural integrity that is necessary for cell adhesion and growth.

The ion-release profiles demonstrate that laponite-containing scaffolds provide a sustained and composition-dependent source of Mg^2+^ over the 21-day incubation period. This behavior is consistent with the dissolution mechanism of laponite and supports its role as an active inorganic component within hybrid biomaterials. The markedly higher ion concentrations observed in the LAP1 formulations indicate that increasing laponite content enhances ionic availability. This controlled Mg^2+^ release is particularly relevant for bone tissue engineering, as magnesium ions play an active role in regulating osteogenic signaling pathways. Previous studies have shown that Mg^2+^ can activate the mechanosensitive transient receptor potential cation channel TRPM7 ion channel and kinase in osteoblasts, triggering osteogenic proliferation and differentiation through downstream phosphatidylinositol 3-kinase (PI3K)-dependent mechanisms [63]. Although the present work did not isolate specific ion-driven pathways, the clear concentration-dependent release trends suggest that Mg^2+^ delivery from laponite could synergize with the polymeric matrix to enhance the osteoinductive potential.

Biologically, all formulations demonstrated intrinsic cytocompatibility, supporting early adhesion and viability of MC3T3-E1 pre-osteoblasts. However, LAP-containing composites outperformed PVA/GEL and carrageenan-containing variants across the functional biological assessment. Specifically in morphological observations, carrageenan incorporation promoted a more uniform distribution of adherent cells, whereas laponite addition led to denser and more elongated cell layers, consistent with stronger focal adhesion and cytoskeletal organization. This observation aligns with previously reported findings [52], indicating that laponite-based polymer nanocomposites enhance cell attachment and spreading through improved surface charge distribution and nanoscale roughness. Over 7 days, metabolic activity and viability increased across all groups, with scaffolds containing 0.5 wt% LAP significantly outperforming 1 wt% LAP, suggesting that moderate nanoclay content better supports early proliferation. Interestingly, while other studies reported on enhanced proliferation at higher LAP concentrations, using similar doses [64], this discrepancy may arise from differences in the polymeric matrices, crosslinking strategies, or scaffold microstructure, all of which govern ion release, porosity, and cell–matrix interactions. In our system, the denser and stiffer network formed at 1 wt% LAP may have partially hindered nutrient diffusion and cell spreading during early culture, outweighing the potential benefits of increased bioactive ion release. These observations highlight the importance of matrix-specific optimization when integrating nanofillers such as laponite into scaffold formulations. The relatively higher standard deviations observed in the cytotoxicity and proliferation assays may be attributed to the inherent heterogeneity of 3D porous scaffolds and variability in initial cell number at each well.

Markers of osteogenic differentiation followed a composition-dependent trend. LAP groups exhibited significantly higher ALP activity than the PVA/GEL control on day 14, reflecting early osteogenic commitment. Also, ALP activity levels were significantly higher in IC-based scaffolds compared to KC-based ones on day 14, suggesting that the higher sulfate content in iota-carrageenan may stimulate differentiation pathways more effectively by modulating the adsorption and conformation of osteogenic proteins from the culture medium such as fibronectin and collagen type I, thereby creating a microenvironment more favorable for early osteogenic signaling and ALP expression [65]. Additionally, between days 14 and 21, LAP-containing composites demonstrated further matrix maturation, as evidenced by increased calcium and collagen production, particularly observed in those containing 1 wt% LAP, confirming a dose-dependent enhancement in mineralization potential and ECM synthesis. Calcium production was markedly elevated in PVA/GEL/KC/LAP1 and PVA/GEL/IC/LAP1 scaffolds on day 21, emphasizing the role of LAP in promoting mineral secretion, consistent with previous reports showing that the release of bioactive silicate ions from laponite activates the osteogenic pathway, through upregulation of RUNX2, thereby enhancing downstream mineralization processes [22,23]. Similarly, collagen synthesis was strongly upregulated in all LAP groups, with LAP1 scaffolds showing significantly higher levels than LAP0.5 across all time points. While no consistent trend was observed between KC and IC, the results underscore the dominant effect of LAP content at this late stage. However, it is worth noting that the maximum collagen concentration in our system was approximately 350 μg/mL, and although it remains lower than those reported in other osteogenic scaffolds (e.g., around 1000 μg/mL in [66]), it is comparable to values reported in hydroxyapatite-based scaffolds [67]. Nevertheless, the results clearly illustrate the synergistic interaction between laponite and carrageenan, consistent with previous studies reporting strong physicochemical compatibility and enhanced structural stability in carrageenan-laponite nanocomposite hydrogels, where electrostatic and hydrogen bonding interactions between the two components improved crosslinking density and structural integrity [29,68]. These molecular interactions align with prior evidence showing that silicate ions contribute to collagen fibrillogenesis and integrin-mediated adhesion, thereby promoting mineralization and extracellular matrix formation [22,69].

Overall, the findings of this study demonstrate a correlation between molecular composition, structural organization, and resulting scaffold performance. The comparative analysis between kappa- and iota-carrageenan revealed that the lower sulfation degree of KC produced a denser and mechanically stronger network, characterized by significantly lower porosity, higher apparent density, and an increased Young’s modulus relative to IC. This structure correlated with higher cell viability and enhanced calcium secretion at later stages, highlighting the contribution of mechanical stability to mineralization. Conversely, IC, with its higher degree of sulfation, formed a more hydrated and open network, which favored protein adsorption and higher ALP activity, suggesting a link between sulfate-mediated bioactivity and early differentiation. The effect of laponite concentration was equally evident. Specifically, scaffolds with LAP0.5 preserved porosity and supported early proliferation, whereas scaffolds with LAP1 led to a notable densification of the pore walls, enhancing matrix mineralization and collagen production, reflecting the reinforcement effect of the higher nanoclay concentration. These results confirm that scaffolds’ composition and architecture impact their biological functionality; by tuning the scaffolds’ composition through variation in the carrageenan type of different sulfation degree, and the laponite content, a relevant structure-property-function correlation can be established. In addition, controlling the crosslinking conditions would lead to a more comprehensive understanding of how molecular design governs the mechanical and osteogenic performance in composite scaffolds for bone tissue engineering.

## 5. Conclusions

We demonstrated that incorporating kappa- or iota-carrageenan and laponite into PVA/GEL scaffolds provides a balanced strategy to couple hydration and porosity with mechanical stability and bioactivity. Carrageenan primarily enhanced swelling and openness of the pore network, supporting cell adhesion, while laponite reinforced the polymer matrix and delivered bioactive ions that promoted osteogenic differentiation. Direct comparison between kappa- and iota-carrageenan did not reveal significant differences in osteogenic performance; however, ΙC-based scaffolds exhibited higher swelling capacity of approximately 574%, whereas KC contributed to marginally higher stiffness, with Young’s modulus values of 109 kPa, due to its lower degree of sulfation and higher density. In contrast, laponite concentration exerted a pronounced effect, with LAP0.5 scaffolds indicating an optimal balance between biocompatibility and proliferation, while LAP1 scaffolds enhanced mechanical strength, yielding Young’s modulus values higher than 225 kPa compared to the LAP0.5 counterparts. Importantly, LAP1 scaffolds exhibited a significant increase in osteogenic differentiation, as evidenced by collagen production of approximately 352 µg/mL. Overall, our findings validate the initial hypothesis that the degree of sulfation in carrageenan affects scaffold performance, regulating hydration and stability, while laponite concentration controls cytocompatibility and osteoinductive response, as evidenced by the significant increase in collagen production in the LAP1 scaffolds compared to LAP0.5. Although non-significant differences were observed between KC- and IC-based formulations in specific biological assays, both systems proved suitable for supporting osteogenic processes. These results suggest that tuning the ratio of natural polysaccharides and inorganic nanoclay is a powerful strategy for designing multifunctional scaffolds that mimic the composite nature of bone tissue.

## Figures and Tables

**Table 1:** Acronyms and compositions of the developed scaffolds.

Scaffold Acronyms	Composition
PVA/GEL	3% *w*/*v* PVA, 3% *w*/*v* gelatin, crosslinked with 0.25% *w*/*v* glutaraldehyde.
PVA/GEL/KC	3% *w*/*v* PVA, 3% *w*/*v* gelatin, 1% *w*/*v* kappa-carrageenan, crosslinked with 0.25% *w*/*v* glutaraldehyde and 0.2 M KCl.
PVA/GEL/IC	3% *w*/*v* PVA, 3% *w*/*v* gelatin, 1% *w*/*v* iota-carrageenan, crosslinked with 0.25% *w*/*v* glutaraldehyde and 0.2 M KCl.
PVA/GEL/KC/LAP0.5	3% *w*/*v* PVA, 3% *w*/*v* gelatin, 1% *w*/*v* kappa-carrageenan, 0.5% *w*/*v* laponite, crosslinked with 0.25% *w*/*v* glutaraldehyde and 0.2 M KCl.
PVA/GEL/IC/LAP0.5	3% *w*/*v* PVA, 3% *w*/*v* gelatin, 1% *w*/*v* iota-carrageenan, 0.5% *w*/*v* laponite, crosslinked with 0.25% *w*/*v* glutaraldehyde and 0.2 M KCl.
PVA/GEL/KC/LAP1	3% *w*/*v* PVA, 3% *w*/*v* gelatin, 1% *w*/*v* kappa-carrageenan, 1% *w*/*v* laponite, crosslinked with 0.25% *w*/*v* glutaraldehyde and 0.2 M KCl.
PVA/GEL/IC/LAP1	3% *w*/*v* PVA, 3% *w*/*v* gelatin, 1% *w*/*v* iota-carrageenan, 1% *w*/*v* laponite, crosslinked with 0.25% *w*/*v* glutaraldehyde and 0.2 M KCl.

**Table 2:** Elemental composition (mass%) monitored by EDS of scaffold cross-sections.

Scaffold Acronyms	C	O	Cl	K	Mg	Si	S
PVA/GEL	58%	40%	1%	1%			
PVA/GEL/KC	55%	40%	2%	3%			
PVA/GEL/IC	55%	42%	1%	2%			
PVA/GEL/KC/LAP0.5	53%	40%	1%	2%	1%	2%	
PVA/GEL/IC/LAP0.5	51%	42%	1%	2%	1%	2%	1%
PVA/GEL/KC/LAP1	53%	39%	1%	2%	2%	2%	1%
PVA/GEL/IC/LAP1	49%	42%	1%	2%	2%	3%	1%

## Data Availability

The original contributions presented in this study are included in the article. Further inquiries can be directed to the corresponding author.

## References

[1] Skalak R. Tissue engineering. Proceedings of the 15th International Conference on IEEE Engineering in Medicine and Biology Society.

[2] O’brien F.J. (2011). Biomaterials & scaffolds for tissue engineering. Mater. Today.

[3] Chen Q.Z., Thompson I.D., Boccaccini A.R. (2006). 45S5 Bioglass^®^-derived glass–ceramic scaffolds for bone tissue engineering. Biomaterials.

[4] Georgopoulou A., Papadogiannis F., Batsali A., Marakis J., Alpantaki K., Eliopoulos A.G., Pontikoglou C., Chatzinikolaidou M. (2018). Chitosan/gelatin scaffolds support bone regeneration. J. Mater. Sci. Mater. Med..

[5] Santoro M., Tatara A.M., Mikos A.G. (2014). Gelatin carriers for drug and cell delivery in tissue engineering. J. Control. Release.

[6] Liu X., Smith L.A., Hu J., Ma P.X. (2009). Biomimetic nanofibrous gelatin/apatite composite scaffolds for bone tissue engineering. Biomaterials.

[7] Campo V.L., Kawano D.F., da Silva D.B., Carvalho I. (2009). Carrageenans: Biological properties, chemical modifications and structural analysis–A review. Carbohydr. Polym..

[8] Yegappan R., Selvaprithiviraj V., Amirthalingam S., Jayakumar R. (2018). Carrageenan based hydrogels for drug delivery, tissue engineering and wound healing. Carbohydr. Polym..

[9] Akram A., Iqbal M., Yasin A., Zhang K., Li J. (2024). Sulfonated molecules and their latest applications in the field of biomaterials: A review. Coatings.

[10] Loukelis K., Papadogianni D., Chatzinikolaidou M. (2022). Kappa-carrageenan/chitosan/gelatin scaffolds enriched with potassium chloride for bone tissue engineering. Int. J. Biol. Macromol..

[11] Rode M.P., Batti Angulski A.B., Gomes F.A., da Silva M.M., Jeremias T.d.S., de Carvalho R.G., Iucif Vieira D.G., Oliveira L.F.C., Fernandes Maia L., Trentin A.G. (2018). Carrageenan hydrogel as a scaffold for skin-derived multipotent stromal cells delivery. J. Biomater. Appl..

[12] Gashti M.P., Stir M., Hulliger J. (2013). Synthesis of bone-like micro-porous calcium phosphate/iota-carrageenan composites by gel diffusion. Colloids Surf. B Biointerfaces.

[13] Neamtu B., Barbu A., Negrea M.O., Berghea-Neamțu C.Ș., Popescu D., Zăhan M., Mireșan V. (2022). Carrageenan-based compounds as wound healing materials. Int. J. Mol. Sci..

[14] Hermansson A.-M., Eriksson E., Jordansson E. (1991). Effects of potassium, sodium and calcium on the microstructure and rheological behaviour of kappa-carrageenan gels. Carbohydr. Polym..

[15] Hassan C.M., Peppas N.A. (2000). Structure and applications of poly (vinyl alcohol) hydrogels produced by conventional crosslinking or by freezing/thawing methods. Biopolymers PVA Hydrogels, Anionic Polymerisation Nanocomposites.

[16] Loukelis K., Kontogianni G.I., Vlassopoulos D., Chatzinikolaidou M. (2025). Extrusion-Based 3D Bioprinted Gellan Gum/Poly (vinyl alcohol)/Nano-Hydroxyapatite Composite Bioinks Promote Bone Regeneration. Adv. Healthc. Mater..

[17] Wei G., Ma P.X. (2004). Structure and properties of nano-hydroxyapatite/polymer composite scaffolds for bone tissue engineering. Biomaterials.

[18] Knaack D., Goad M., Aiolova M., Rey C., Tofighi A., Chakravarthy P., Lee D.D. (1998). Resorbable calcium phosphate bone substitute. J. Biomed. Mater. Res..

[19] Loukelis K., Helal Z.A., Mikos A.G., Chatzinikolaidou M. (2023). Nanocomposite bioprinting for tissue engineering applications. Gels.

[20] Dawson J.I., Oreffo R.O. (2013). Clay: New opportunities for tissue regeneration and biomaterial design. Adv. Mater..

[21] Tomás H., Alves C.S., Rodrigues J. (2018). Laponite^®^: A key nanoplatform for biomedical applications?. Nanomed. Nanotechnol. Biol. Med..

[22] Gaharwar A.K., Mihaila S.M., Swami A., Patel A., Sant S., Reis R.L., Marques A.P., Gomes M.E., Khademhosseini A. (2023). Bioactive silicate nanoplatelets for osteogenic differentiation of human mesenchymal stem cells. Adv. Mater..

[23] Li T., Liu Z.L., Xiao M., Yang Z.Z., Peng M.Z., Li C.D., Zhou X.J., Wang J.W. (2018). Impact of bone marrow mesenchymal stem cell immunomodulation on the osteogenic effects of laponite. Stem Cell Res. Ther..

[24] Li J., Yang B., Qian Y., Wang Q., Han R., Hao T., Shu Y., Zhang Y., Yao F., Wang C. (2015). Iota-carrageenan/chitosan/gelatin scaffold for the osteogenic differentiation of adipose-derived MSCs in vitro. J. Biomed. Mater. Res. Part B Appl. Biomater..

[25] Pettinelli N., Rodriguez-Llamazares S., Bouza R., Barral L., Feijoo-Bandin S., Lago F. (2020). Carrageenan-based physically crosslinked injectable hydrogel for wound healing and tissue repairing applications. Int. J. Pharm..

[26] Bui V.T., Nguyen B.T., Nicolai T., Renou F. (2019). Mobility of carrageenan chains in iota-and kappa carrageenan gels. Colloids Surf. A Physicochem. Eng. Asp..

[27] Geonzon L.C., Descallar F.B.A., Du L., Bacabac R.G., Matsukawa S. (2020). Gelation mechanism and network structure in gels of carrageenans and their mixtures viewed at different length scales–A review. Food Hydrocoll..

[28] Rhim J.-W., Wang L.-F. (2014). Preparation and characterization of carrageenan-based nanocomposite films reinforced with clay mineral and silver nanoparticles. Appl. Clay Sci..

[29] Papagiannopoulos A., Nikolakis S.-P., Pamvouxoglou A., Koutsopoulou E. (2023). Physicochemical properties of electrostatically crosslinked carrageenan/chitosan hydrogels and carrageenan/chitosan/Laponite nanocomposite hydrogels. Int. J. Biol. Macromol..

[30] Sears C., Mondragon E., Richards Z.I., Sears N., Chimene D., McNeill E.P., Gregory C.A., Gaharwar A.K., Kaunas R. (2020). Conditioning of 3D printed nanoengineered ionic–covalent entanglement scaffolds with iP-hMSCs derived matrix. Adv. Healthc. Mater..

[31] Sarkar B.R., Chauhan U. (1967). A new method for determining micro quantities of calcium in biological materials. Anal. Biochem..

[32] Ding S.-J., Wei C.-K., Lai M.-H. (2011). Bio-inspired calcium silicate–gelatin bone grafts for load-bearing applications. J. Mater. Chem..

[33] Zhang R., Ma P.X. (1999). Poly (α-hydroxyl acids)/hydroxyapatite porous composites for bone-tissue engineering. I. Preparation and morphology. J. Biomed. Mater. Res. Off. J. Soc. Biomater. Jpn. Soc. Biomater. Aust. Soc. Biomater..

[34] Vimalraj S. (2020). Alkaline phosphatase: Structure, expression and its function in bone mineralization. Gene.

[35] Hadjicharalambous C., Kozlova D., Sokolova V., Epple M., Chatzinikolaidou M. (2015). Calcium phosphate nanoparticles carrying BMP-7 plasmid DNA induce an osteogenic response in MC3T3-E1 pre-osteoblasts. J. Biomed. Mater. Res. Part A.

[36] Hadjicharalambous C., Mygdali E., Prymak O., Buyakov A., Kulkov S., Chatzinikolaidou M. (2015). Proliferation and osteogenic response of MC 3 T 3-E1 pre-osteoblastic cells on porous zirconia ceramics stabilized with magnesia or yttria. J. Biomed. Mater. Res. Part A.

[37] Gómez-Ordóñez E., Rupérez P. (2011). FTIR-ATR spectroscopy as a tool for polysaccharide identification in edible brown and red seaweeds. Food Hydrocoll..

[38] Webber V., Carvalho S.M.d., Ogliari P.J., Hayashi L., Barreto P.L.M. (2012). Optimization of the extraction of carrageenan from Kappaphycus alvarezii using response surface methodology. Food Sci. Technol..

[39] Mihaila S.M., Gaharwar A.K., Reis R.L., Marques A.P., Gomes M.E., Khademhosseini A. (2013). Photocrosslinkable kappa-carrageenan hydrogels for tissue engineering applications. Adv. Healthc. Mater..

[40] Skelton S., Bostwick M., O’Connor K., Konst S., Casey S., Lee B.P. (2013). Biomimetic adhesive containing nanocomposite hydrogel with enhanced materials properties. Soft Matter.

[41] Barbosa M.L., Oliveira L.M.d., Paiva R., Dametto A.C., Dias D.d.S., Ribeiro C.A., Wrona M., Nerín C., Barud H.d.S., Cruz S.A. (2023). Evaluation the potential of onion/laponite composites films for sustainable food packaging with enhanced UV protection and antioxidant capacity. Molecules.

[42] Vashisth P., Nikhil K., Roy P., Pruthi P.A., Singh R.P., Pruthi V. (2016). A novel gellan–PVA nanofibrous scaffold for skin tissue regeneration: Fabrication and characterization. Carbohydr. Polym..

[43] Das M.P., Suguna P., Prasad K., Vijaylakshmi J., Renuka M. (2017). Extraction and characterization of gelatin: A functional biopolymer. Int. J. Pharm. Pharm. Sci.

[44] Croitoru C., Pop M.A., Bedo T., Cosnita M., Roata I.C., Hulka I. (2020). Physically crosslinked poly (vinyl alcohol)/kappa-carrageenan hydrogels: Structure and applications. Polymers.

[45] Ghani N.A.A., Othaman R., Ahmad A., Anuar F.H., Hassan N.H. (2019). Impact of purification on iota carrageenan as solid polymer electrolyte. Arab. J. Chem..

[46] Liu Z., Zhao Z., Jin X., Wang L.-M., Liu Y.D. (2021). Preparation of cellulose/laponite composite particles and their enhanced electrorheological responses. Molecules.

[47] Daniel L.M., Frost R.L., Zhu H.Y. (2008). Edge-modification of laponite with dimethyl-octylmethoxysilane. J. Colloid Interface Sci..

[48] Jafari A., Vahid Niknezhad S., Kaviani M., Saleh W., Wong N., Van Vliet P.P., Moraes C., Ajji A., Kadem L., Azarpira N. (2024). Formulation and Evaluation of PVA/Gelatin/Carrageenan Inks for 3D Printing and Development of Tissue-Engineered Heart Valves. Adv. Funct. Mater..

[49] Mao J.S., Zhao L.G., Yin Y.J., De Yao K. (2003). Structure and properties of bilayer chitosan–gelatin scaffolds. Biomaterials.

[50] Huang Y., Onyeri S., Siewe M., Moshfeghian A., Madihally S.V. (2005). In vitro characterization of chitosan–gelatin scaffolds for tissue engineering. Biomaterials.

[51] Yu C., Dou X., Meng L., Feng X., Gao C., Chen F., Tang X. (2023). Structure, rheological properties, and biocompatibility of Laponite^®^ cross-linked starch/polyvinyl alcohol hydrogels. Int. J. Biol. Macromol..

[52] Ghadiri M., Chrzanowski W., Lee W., Fathi A., Dehghani F., Rohanizadeh R. (2013). Physico-chemical, mechanical and cytotoxicity characterizations of Laponite^®^/alginate nanocomposite. Appl. Clay Sci..

[53] Karageorgiou V., Kaplan D. (2005). Porosity of 3D biomaterial scaffolds and osteogenesis. Biomaterials.

[54] Geonzon L.C., Bacabac R.G., Matsukawa S. (2019). Network structure and gelation mechanism of kappa and iota carrageenan elucidated by multiple particle tracking. Food Hydrocoll..

[55] Pawde S., Deshmukh K. (2008). Characterization of polyvinyl alcohol/gelatin blend hydrogel films for biomedical applications. J. Appl. Polym. Sci..

[56] Pasini C., Re F., Trenta F., Russo D., Sartore L. (2024). Gelatin-Based Scaffolds with Carrageenan and Chitosan for Soft Tissue Regeneration. Gels.

[57] Kanungo B.P., Gibson L.J. (2010). Density–property relationships in collagen–glycosaminoglycan scaffolds. Acta Biomater..

[58] Gerhardt L.-C., Boccaccini A.R. (2010). Bioactive glass and glass-ceramic scaffolds for bone tissue engineering. Materials.

[59] Hwang H.S., Lee C.-S. (2024). Nanoclay-composite hydrogels for bone tissue engineering. Gels.

[60] Hilliou L. (2021). Structure–elastic properties relationships in gelling carrageenans. Polymers.

[61] Haraguchi K., Takehisa T. (2002). Nanocomposite hydrogels: A unique organic–inorganic network structure with extraordinary mechanical, optical, and swelling/de-swelling properties. Adv. Mater..

[62] Sivakumar P.M., Yetisgin A.A., Sahin S.B., Demir E., Cetinel S. (2022). Bone tissue engineering: Anionic polysaccharides as promising scaffolds. Carbohydr. Polym..

[63] Zhang X., Zu H., Zhao D., Yang K., Tian S., Yu X., Lu F., Liu B., Yu X., Wang B. (2017). Ion channel functional protein kinase TRPM7 regulates Mg ions to promote the osteoinduction of human osteoblast via PI3K pathway: In vitro simulation of the bone-repairing effect of Mg-based alloy implant. Acta Biomater..

[64] Mignon A., Pezzoli D., Prouvé E., Lévesque L., Arslan A., Pien N., Schaubroeck D., Van Hoorick J., Mantovani D., Van Vlierberghe S. (2019). Combined effect of Laponite and polymer molecular weight on the cell-interactive properties of synthetic PEO-based hydrogels. React. Funct. Polym..

[65] Nourmohammadi J., Roshanfar F., Farokhi M., Nazarpak M.H. (2017). Silk fibroin/kappa-carrageenan composite scaffolds with enhanced biomimetic mineralization for bone regeneration applications. Mater. Sci. Eng. C.

[66] Papadogiannis F., Batsali A., Klontzas M.E., Karabela M., Georgopoulou A., Mantalaris A., Zafeiropoulos N.E., Chatzinikolaidou M., Pontikoglou C. (2020). Osteogenic differentiation of bone marrow mesenchymal stem cells on chitosan/gelatin scaffolds: Gene expression profile and mechanical analysis. Biomed. Mater..

[67] Salifu A.A., Lekakou C., Labeed F.H. (2017). Electrospun oriented gelatin-hydroxyapatite fiber scaffolds for bone tissue engineering. J. Biomed. Mater. Res. Part A.

[68] Mahdavinia G.R., Massoudi A., Baghban A., Massoumi B. (2012). Novel carrageenan-based hydrogel nanocomposites containing laponite RD and their application to remove cationic dye. Iran. Polym. J..

[69] Hoppe A., Güldal N.S., Boccaccini A.R. (2011). A review of the biological response to ionic dissolution products from bioactive glasses and glass-ceramics. Biomaterials.

